# Impact of prior malignancies on outcome of colorectal cancer; revisiting clinical trial eligibility criteria

**DOI:** 10.1186/s12885-019-6074-6

**Published:** 2019-08-30

**Authors:** Muneer J. Al-Husseini, Anas M. Saad, Hadeer H. Mohamed, Mohamad A. Alkhayat, Mohamad Bassam Sonbol, Omar Abdel-Rahman

**Affiliations:** 1 Department of Medicine, Ascension St John Hospital, Detroit, MI USA; 2 Clinical Oncology, Faculty of Medicine, Damascus University, Fayez Mansour Street, Damascus, Syria; 3 Department of Oncology, Faculty of Medicine, Ain Shams University, Cairo, Egypt; 4 Faculty of Medicine, Ain Shams University, Cairo, Egypt; 5 Mayo Clinic Cancer Center, Phoenix, AZ USA; 6 Department of Oncology, University of Alberta and Cross Cancer Institute, Edmonton, Alberta Canada; 7 Clinical Oncology department, Faculty of Medicine, Ain Shams University, Lofty Elsayed Street, Cairo, 11566 Egypt

**Keywords:** Colorectal cancer, Prior malignancy, SEER database, Clinical trials, Eligibility, Survival analysis

## Abstract

**Background:**

Most clinical trials on colorectal cancer (CRC) exclude cases who have history of a prior malignancy. However, no prior research studied this history’s actual impact on the survival of CRC. In the paper, we study the effects of having a malignancy preceding CRC diagnosis on its survival outcomes.

**Methods:**

CRC patients diagnosed during 1973–2008 were reviewed using the SEER 18 database. We calculated overall survival and cancer-specific survival of subsequent CRC, and more specifically stage IV CRC, using Kaplan-Meier test and adjusted Cox models.

**Results:**

A total 550,325 CRC patients were reviewed, of whom 31,663 had history of a prior malignancy. The most commonly reported sites of a prior malignancy were: prostate, breast, urinary bladder, lung, and endometrium. Patients with history of a prior non-leukemic malignancy or history of a prior leukemia were found to have worse overall survival (HR = 1.165 95%CI = 1.148–1.183, *P* < 0.001) and (HR = 1.825 95%CI = 1.691–1.970, *P* < 0.001), respectively. However, CRC patients with history of a prior non-leukemic malignancy showed an improved colorectal cancer-specific survival (HR = .930 95%CI = .909–.952, *P* < 0.001). Analysis of stage IV CRC patients showed that patients with history of any non-leukemic malignancy did not have a significant change in overall survival. Whereas, patients with a prior leukemia showed a worse overall survival (HR = 1.535, 95%CI = 1.303–1.809, *P* < 0.001). When analyzed separately, right CRC and left CRC showed similar survival patterns.

**Conclusion:**

A prior malignancy before CRC -in general- can be associated with worse clinical survival outcomes. These worse outcomes are not observed in stage IV CRC. Considering these results when including/excluding stage IV CRC patients with prior malignancies in clinical trials may play help improve their generalizability.

**Electronic supplementary material:**

The online version of this article (10.1186/s12885-019-6074-6) contains supplementary material, which is available to authorized users.

## Background

Colorectal cancer (CRC) is the third most common cancer in men and women in the US. It is expected that 101,420 cases of CRC will occur in 2019. CRC ranks third in terms of mortality in both men and women with the mean age of CRC diagnosis being 68 years [1–3].

Among individuals older than 50 years, incidence rates of CRC have been decreasing starting from the mid-1980s reaching a progressive decline in the past 5 years. However, the incidence has risen in younger populations by 22%. The previously mentioned decline can be linked to the modification of CRC risk factors like the decrease in the number of tobacco smokers and the use of non-steroidal anti-inflammatory drugs [4]. Mortality rates have been dropping in both sexes with an overall decline of 49% between 1976 and 2012. According to American cancer society colorectal cancer statistics 2017, the 5-year relative survival rate for CRC patients diagnosed from 2006 to 2012 was 65% [2].

Patients with a history of cancer are not usually well-represented in clinical trials with most of the studies considering a “history of cancer” as an exclusion criterion. This practice can potentially impact the accrual of these trials and limit possible therapies for this population. The rationale behind this exclusion is the assumption that a prior malignancy may affect the study outcomes [5]. Lichtman et al. have studied this phenomenon and concluded that inclusion of patients with prior malignancies in trials is recommended especially if the prior malignancy does not interfere with either efficacy or safety endpoints. Also, if treatment of the prior malignancy was finished 2 years before joining the clinical trial [6]. Accordingly, we used data from the Surveillance, Epidemiology, and End Results (SEER) program of the US National Cancer Institute to report the impact of a history of prior cancer on the survival of subsequent colorectal cancer in general, and stage IV CRC in particular. Moreover, we aimed at studying the latency period between the first malignancy and CRC after which joining clinical trials does not adversely impact CRC patients’ survival outcomes to give evidence for the eligibility of enrollment of CRC patients in clinical trials.

## Methods

### Data sources

We obtained approval to use the SEER database, using the SEER*stat software (version 8.3.3) [7]. We used the SEER 18 Registries, Nov 2015 Submission (1973–2013 varying), which cover about 27.8% of the U. S general population [8].

### Study design

We performed a retrospective cohort study, according to the guidelines of the STROBE (Strengthening the Reporting of Observational Studies in Epidemiology Statements) checklist [9].

### Study population

We selected patients with CRC (Site Recode ICD-O-3/WHO codes: C180-C189, C199, C209, C260) diagnosed between 1973 and 2008. Cases whose diagnosis was only based on death certificates or autopsy were excluded. We checked the history of each patient for having a previous registry of a diagnosis of another primary malignancy. The sample was then divided into two groups according to having a history of a prior diagnosis of malignancy or not. To be able to eliminate the probability of simultaneous cancers, we excluded patients whose colorectal malignancies were diagnosed during the 6 months following the first malignancy’s diagnosis. We also excluded patients whose prior malignancy was CRC. Patients who had more than one primary malignancy (other than CRC) were excluded.

Then the group with a prior malignancy was subdivided based on the site of the prior cancer. Solid cancers generally all followed the same trend in survival outcomes, whereas a history of leukemia followed a significantly different trend in survival. We, therefore, grouped patients and reported survival outcomes based having a prior history of leukemia or a non-leukemic malignancy. In order to be able to assess different survival outcomes according to the latency period between the two diagnoses, which is important to answer the question of clinical trials exclusion criteria, we also stratified the group with a prior malignancy according to the latency period between the two diagnoses: patients developing CRC within 7–12 months of the prior malignancy, patients developing CRC within 1–5 years after the prior malignancy and, patients developing CRC 5 years or more after the prior malignancy.

We obtained information of the following variables in included patients: race, sex, marital status at the diagnosis of the colorectal cancer, date of diagnosis, age at diagnosis, site of cancer, histology of cancer, stage of cancer (based on SEER historic stage A), grade of cancer, exposure to radiation for treatment, prior surgery (or ablation) for treatment, survival months, vital state, and the cause of death. Patients with missing data for sex, age, and the date of diagnosis of either malignancy were excluded. Patients with a missing date for other variables were only excluded in Cox regression models.

### Outcomes

We calculated two main outcomes: overall survival and colorectal cancer-specific survival. Survival was defined as the interval in months between diagnosis and death. Patients were followed until dates of death or censored at the end of 2013. In the case of colorectal cancer-specific survival, patients were censored if death occurred because of any cause other than colorectal cancer. We assessed these outcomes in the study groups to study the effects of a prior malignancy on both the survival of the colorectal malignancy.

To evaluate the impact of a prior malignancy on the survival outcomes of stage IV CRC and answer the question of clinical trials’ exclusion criteria, we conducted a further analysis by selecting only patients with stage IV CRC and measured the overall survival and colorectal cancer-specific survival.

Furthermore, we analyzed the impact of a prior history of malignancy on the survival of left colon cancer (LCRC), right colon cancer (RCRC) separately; as multiple studies have correlated the side of the origin of colon cancer to its survival [9, 10]. LCRC was defined as a CRC in any of the following sites: descending colon, sigmoid colon, or rectum. RCRC was defined as CRC originating from the cecum or ascending colon. Malignancies of the transverse colon and the appendix were excluded from this subgroup analysis. We also excluded cases with an unknown specific site in the large intestine from this subgroup analysis. We went a further step comparing the overall survival of stage IV LCRC and stage IV RCRC.

### Statistical analysis

We used SEER*stat software to query patients’ data from the SEER database, and we used SPSS software (version 23, IBM, NY) to perform all the analyses mentioned in the manuscript except for the competing risk analysis. We constructed Kaplan-Meier survival curves according to the presence/absence of a prior malignancy and performed log-rank tests, and multivariable covariate-adjusted Cox regression to perform the previously mentioned survival tests. We adjusted for the following factors: the presence of a prior malignancy, age at diagnosis of CRC, sex, race, marital status, the stage of CRC, the grade of CRC, radiation, and surgery as treatment options for CRC. We further did a competing risk analysis to assess colorectal cancer-specific survival and the effects of colorectal cancer versus other causes of death on the survival of patients, with adjustment for the presence of a prior malignancy, age at diagnosis of CRC, sex, race, marital status, stage of CRC, grade of CRC, radiation and surgery as treatment options for CRC. We used STATA 14.2 software for the competing risk analysis. All statistical tests were two-sided. A *p*-value of less than .05 was considered significant.

## Results

### Patients’ characteristics

**Fig. 1:**
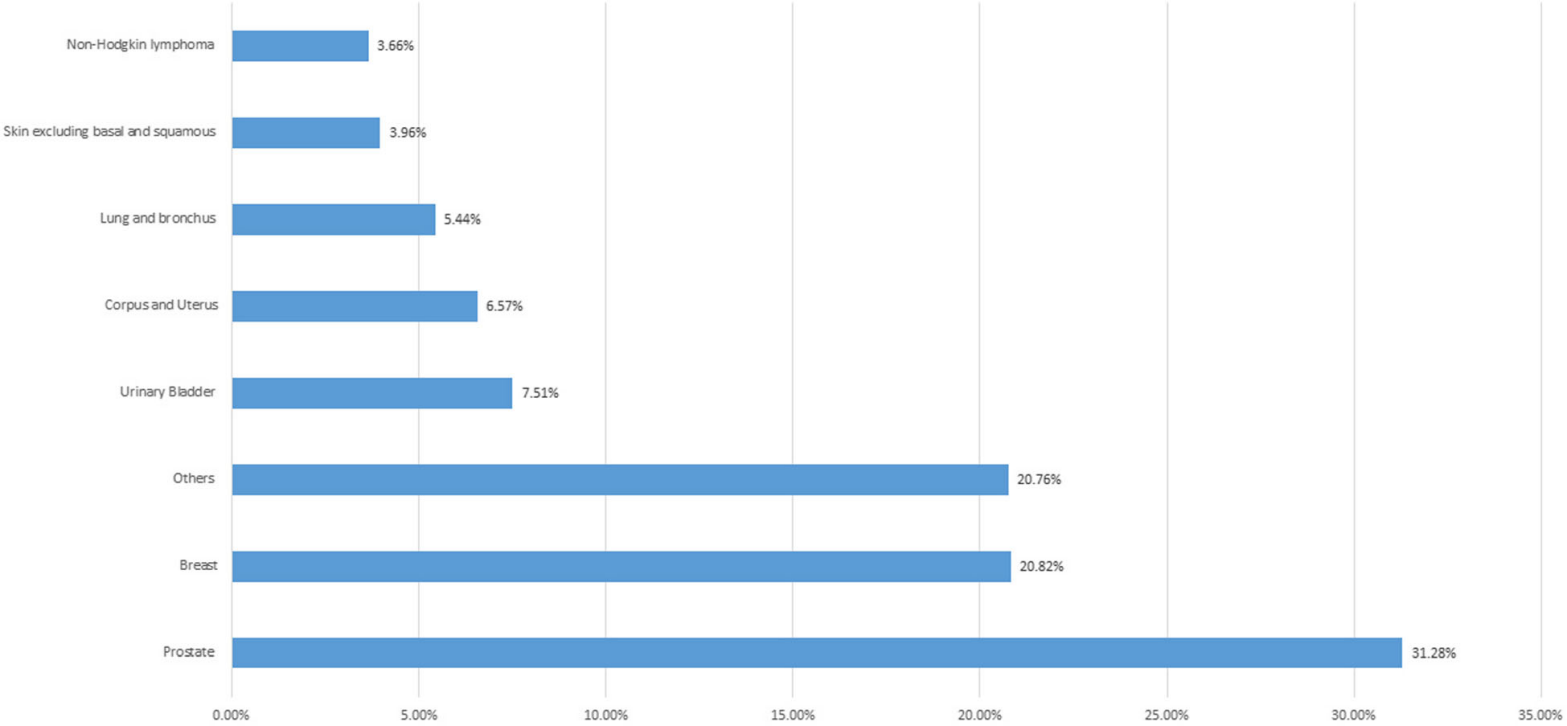
Illustrates the sites of prior malignancies before CRC diagnosis

A total of 550,325 patients with colorectal cancer were reviewed of whom 31,663 (5.8%) had a prior malignancy. Baseline characteristics of the sample are listed in Table 1. The most common sites were: prostate (31.28%), breast (20.82%), and urinary bladder (7.51%). In males, the most common cancers were: prostate (56.5%), urinary bladder (10.4%), and lung (5.7%); whereas among females, breast (46.3%), endometrium (14.7%), and lung (5.1%) were the most commonly reported cancers. Figure 1 shows the commonest sites of prior malignancies preceding CRC diagnosis.

**Table 1:** Baseline patient characteristics of the colorectal cancer cohort (*n* = 550,325)

Patient Characteristics	All patients No.	Prior malignancy No. (%)^+^	No prior malignancy No. (%)^+^
Age			
< 20	319	2 (0.6)	317 (99.4)
20–65	190507	5091 (2.7)	185416 (97.3)
> 65	359499	26570 (7.4)	332929 (92.6)
Sex			
Male	271959	17535 (6.4)	254424 (93.6)
Female	278366	14128 (5.1)	264238 (94.9)
Site			
Rectum	161326	8224 (5.1)	153102 (94.9)
Colon	388999	23439 (6)	365560 (94.0)
Race			
White	458185	27267 (6)	430918 (94.0)
Black	52918	2845 (5.4)	50073 (94.6)
Others	36920	1538 (4.2)	35382 (95.8)
Marital Status			
Single	57126	2470 (4.3)	54656 (95.7)
Married	299526	17787 (5.9)	281739 (94.1)
Separated	8217	282 (3.4)	7935 (96.6)
Divorced	37197	1891 (5.1)	35306 (94.9)
Widowed	126147	8086 (6.4)	118061 (93.6)
Stage			
Localized	199159	12848 (6.5)	186311 (93.5)
Regional	197018	10884 (5.5)	186134 (94.5)
Distant	119763	5980 (5)	113783 (95.0)
Grade			
Well differentiated	53999	3032 (5.6)	50967 (94.4)
Moderately differentiated	288514	17377 (6)	271137 (94.0)
Poorly differentiated	88748	5470 (6.2)	83278 (93.8)
Undifferentiated, Anaplastic	5936	374 (6.3)	5562 (93.7)
Histology recode broad groupings			
Adenomas and adenocarcinomas	465847	26782 (5.7)	439065 (94.3)
Cystic, mucinous and serous neoplasms	55884	3426 (6.1)	52458 (93.9)
Epithelial neoplasms, NOS	18108	925 (5.1)	17183 (94.9)
Squamous cell neoplasms	1815	118 (6.5)	1697 (93.5)
Radiation			
Yes	61226	2381 (3.9)	58845 (96.1)
No	482430	29039 (6)	453391 (94.0)
Surgery			
Yes	467713	26940 (5.8)	440773 (94.2)
No	60051	4020 (6.7)	56031 (93.3)
Ablation	807	61 (7.6)	746 (92.4)

^+^This number represents the percentage of patients with a prior malignancy within each characteristic

### Effect of prior malignancy on overall and colorectal cancer-specific survival

**Fig. 2:**
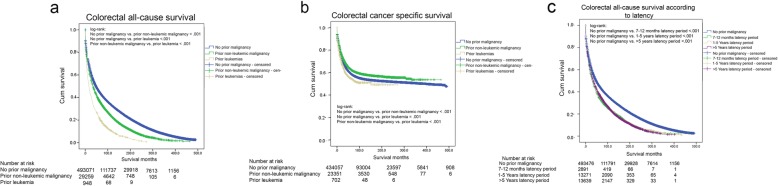
All-cause (**a**) and colorectal cancer-specific (**b**) survival for colon cancer patients with and without prior non-leukemic malignancy / leukemia (**c**) All-cause for CRC patients according to the latency period. All statistical tests were two-sided

Log-rank test on Kaplan Meier curves showed a statistically significant difference in both overall and colorectal-cancer specific survival between patients with prior history of non-leukemic malignancy (median overall survival = 35 months, 95% CI [34.057–35.943]), patients with a prior leukemia (median overall survival = 16 months, 95% CI [13.552–18.448]), and patients with no prior history of malignancy (median overall survival = 48 months, 95% CI [47.642–48.358]) (Fig. 2). When overall survival was analyzed according to the latency period between CRC diagnosis and the prior malignancy, the survival of CRC patients without a prior malignancy was significantly superior to the survival of all latency groups (Fig. 2).

The assumption of proportional hazard for the adjusted Cox model was violated for overall survival (*P* < .001), but it was met for colorectal-cancer specific survival (*P* = .06). Multivariable covariate-adjusted Cox models showed that having a history of any non-leukemic malignancy showed a statistically significant favorable colorectal cancer-specific survival (HR = .930 95% CI = .909–.952, *P* < 0.001) but having a history of leukemia still showed a worse colorectal cancer-specific survival (HR = 1.263, 95% CI = 1.116–1.430, *P* < 0.001) (Table 2). Within this cox model, we studied the interaction over time between having a history of a prior malignancy, and each one of the other adjusted variables (age, sex, race, marital status, stage, grade, radiation, and surgery). None of these variables interacted significantly with having a prior malignancy (data not shown).

**Table 2:** Multivariable covariate-adjusted Cox models for colorectal cancer-specific survival

Patient characteristics	Colorectal cancer–specific HR^a^ (95% CI)^b^	Colorectal cancer–specific *P* value^c^
Prior cancer diagnosis (vs none)		
Prior non-leukemic malignancies	.930 (.909 to .952)	<.001
Prior leukemias	1.263 (1.116 to 1.430)	<.001
Age (vs 20–65), y		
< 20	.931 (.748 to 1.158)	.531
> 65	1.376 (1.361 to 1.392)	<.001
Sex (vs female)		
Male	1.124 (1.112 to 1.136)	<.001
Race (vs white)		
Black	1.135 (1.116 to 1.154)	<.001
Others	.872 (.854 to .890)	<.001
Marital status (vs single)		
Married	.895 (.880 to .910)	<.001
Separated	1.116 (1.066 to 1.168)	<.001
Divorced	.942 (.920 to .965)	<.001
Widowed	1.108 (1.087 to 1.129)	<.001
Stage (VS Localized)		
Regional	2.983 (2.937 to 3.029)	<.001
Distant	13.548 (13.331 to 13.769)	<.001
Grade (Vs Well Differentiated)		
Moderately Differentiated	1.153 (1.132 to 1.174)	<.001
Poorly Differentiated	1.668 (1.634 to 1.701)	<.001
Undifferentiated and Anaplastic	1.904 (1.828 to 1.983)	<.001
Radiation (Vs No)		
Yes	1.019 (1.004 to 1.034)	.011
Surgery (Vs No)		
Yes	.434 (.427 to .442)	<.001
Ablation	1.058 (.918 to 1.219)	.435

^a^this number represent the hazard ratio for all cause and colorectal cancer specific death for the above coverables. All statistical tests were tow-sided

^b^this represents confidence interval

^c^Two-sided *P* value was calculated from multivariable covariate-adjusted Cox models

We further did a competing risk analysis to assess colorectal cancer-specific survival and the effects of colorectal cancer versus other causes of death on the survival of patients. After adjustment for age, sex, race, marital status, stage of CRC, grade of CRC, radiation, and surgery, we found that a non-leukemic malignancy was associated with better colorectal-cancer specific outcomes, with a subhazard ratio (SHR) of .89 (95% CI = .80–.84, *P* < .001). However, having a history of leukemia was no different colorectal cancer-survival outcomes (SHR = .88, 95% CI = .75–1.03, *P* = .107).

The same trends were observed when looking at CRC outcomes based on the site. For both groups, LCRC and RCRC, history of prior non-leukemic malignancy was associated with worse overall survival and favorable colorectal cancer-specific survival. On the other hand, a prior leukemia was associated with inferior overall survival in both LCRC and RCRC groups, and worse cancer-specific survival in LCRC group, but did not negatively impact cancer-specific survival in the RCRC group (Additional file 1).

### Effect of prior malignancy on stage IV CRC patients’ survival

**Fig. 3:**
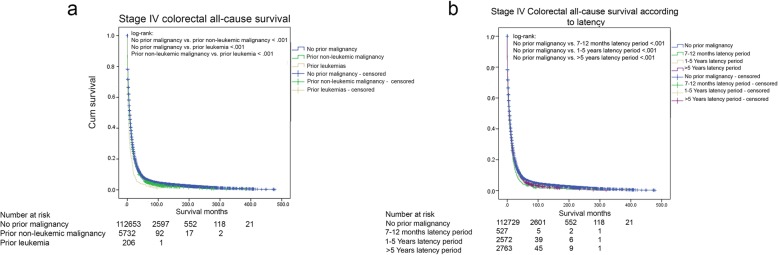
All-cause survival colon for stage IV cancer patients (**a**) with and without prior non-leukemic malignancy / leukemia (**b**) according to the latency period. All statistical tests were two-sided

When studying stage IV CRC patients, log-rank test on Kaplan Meier curves showed a statistically significant difference in overall survival between patients with any prior history of non-leukemic malignancy (median overall survival = 6 months, 95% CI [5.627–6.373]), patients with a prior leukemia (median overall survival = 3 months, 95% CI [2.040–3.960]), and patients with no prior history of malignancy (median overall survival = 8 months, 95% CI [7.904–8.096]), demonstrating the best overall survival when not having a prior malignancy, and the worst overall survival when having a leukemia (Fig. 3).

The assumption of proportional hazard for this cox model was met (*P* = .128), and after adjusting for multiple factors using a multivariable Cox model, having prior non-leukemic malignancy did not impact overall survival (HR = .998, 95% CI = .966–1.031, *P* = 0.907) but having a history of leukemia was associated with a worse overall survival (HR = 1.535, 95% CI = 1.303–1.809, *P* < 0.001) (Table 3). Within this cox model, we studied the interaction over time between having a history of a prior malignancy, and each one of the other adjusted variables (age, sex, race, marital status, grade, radiation, and surgery). None of these variables interacted significantly with having a prior malignancy (data not shown).

**Table 3:** multivariable covariate-adjusted Cox models for overall survival for stage ‘IV’ colorectal cancer

Patient characteristics	Stage IV colorectal All-cause HR^a^(95% CI)^b^	Stage IV All-cause *P value*^c^
Prior cancer diagnosis (vs none)		
Prior non-leukemic malignancies	.998 (.966 to 1.031)	.907
Prior leukemias	1.535 (1.303 to 1.809)	<.001
Age (vs 20–65), y		
< 20	.851 (.644 to 1.124)	.255
> 65	1.452 (1.429 to 1.475)	<.001
Sex (vs female)		
Male	1.060 (1.044 to 1.076)	<.001
Race (vs white)		
Black	1.095 (1.071 to 1.120)	<.001
Others	.892 (.867 to .918)	<.001
Marital status (vs single)		
Married	.920 (.899 to .940)	<.001
Separated	1.092 (1.025 to 1.164)	.007
Divorced	.959 (.929 to .990)	.010
Widowed	1.120 (1.090 to 1.150)	<.001
Grade (Vs Well Differentiated)		
Moderately Differentiated	1.125 (1.094 to 1.157)	<.001
Poorly Differentiated	1.596 (1.550 to 1.644)	<.001
Undifferentiated and Anaplastic	1.833 (1.738 to 1.932)	<.001
Radiation (Vs No)		
Yes	.690 (.674 to .707)	<.001
Surgery (Vs No)		
Yes	.471 (.462 to .480)	<.001
Ablation	.979 (.808 to 1.185)	.825

^a^this number represent the hazard ratio for all cause death for the above coverables. All statistical tests were tow-sided

^b^this represents confidence interval

^c^Two-sided *P* value was calculated from multivariable covariate-adjusted Cox models

When survival was analyzed according to the latency period between CRC diagnosis and the prior malignancy, the survival of stage IV CRC patients without a prior malignancy was significantly superior to the survival of all latency groups (Fig. 3). However, after adjusting for multiple factors using a multivariable Cox model, a prior malignancy was associated with a worse survival only when diagnosed within 7–12 months before stage IV CRC (HR = 1.127, 95% CI = 1.015–1.251, *P* = .025). Whereas, when CRC was diagnosed a year or more after the prior malignancy, the prior malignancy was not associated with a statistically significant difference (data not shown).

### Effect of prior malignancy on stage IV right and left CRC patients

The same trends were observed when analyzing stage IV LCRC and RCRC patients separately. Kaplan Meier curves and log-rank test showed a statistically significant difference in overall survival between patients with any prior history of non-leukemic malignancy, patients with a prior leukemia, and patients with no prior history of malignancy, demonstrating the best overall survival when not having a prior malignancy, and the worst overall survival when having leukemia (Additional file 2).

When we compared the overall survival of stage IV LCRC with stage IV RCRC based on having a history of prior malignancy, we found that stage IV LCRC without a history of a prior malignancy has the best overall survival, followed by stage IV LCRC with a history of a prior malignancy, then stage IV RCRC without a history of a prior malignancy, and the worst survival with stage IV RCRC with a history of a prior malignancy (Additional file 2).

## Discussion

We found that a prior non-leukemic malignancy was not associated with worse overall survival in stage IV CRC patients and better cancer-specific survival in CRC patients in general. Recently, patients with a history of malignancies have been undergoing tighter screening and surveillance [6]. This screening process is mostly performed through colonoscopy or sigmoidoscopy which are invasive, unfavorable by the patients, and expensive. It has been postulated that CRC biomarkers may offer an alternative to invasive screening procedures and can also be used for prognostic and personalized therapeutic purposes [10, 11].

Due to these effective screening measures, the occurrence of subsequent primary malignancies tends to be discovered and managed earlier among these patients compared to others without such history [12]. As a result, it has been hypothesized that this enhanced surveillance can have a positive impact on the survival outcomes of patients with prior cancers [13]. In accordance with this hypothesis, Multivariable covariate-adjusted Cox models showed that patients who had a prior history of non-leukemic malignancy had better colorectal cancer-specific survival compared to patients without a prior malignancy; nevertheless, patients who had a prior history of leukemia still had worse colorectal cancer-specific survival than patients without a prior history of leukemia. These results were further confirmed by studying the interaction between variables in the Cox model that showed no significant interaction between having a prior malignancy and each of the other variables; asserting that the effect of prior malignancy is homogenous across subgroups. However, when we performed a competing-risks to further assess colorectal cancer-specific survival and other causes of death, no significant difference was observed in colorectal cancer-specific survival in the presence of a prior leukemia.

We found that patients with a history of prior malignancy tend to have worse overall survival outcomes. This difference between the effect of a prior malignancy on the overall and cancer-specific survival may be attributed to the censoring of patients who died from the first cancer (during the cancer-specific survival analyses). Furthermore, overall survival data were assessed according to the latency period between diagnosis of CRC and prior malignancy. Survival of CRC patients without a prior malignancy was significantly better than the survival of all latency groups, with patients who developed CRC within 7–12 months prior to CRC having the worst survival.

A previous British single-center study on gastrointestinal cancers showed that the stage of the second cancer is the main predictor of its survival and a prior history of cancer is unlikely to be relevant to the second cancer’s survival [14]. Another SEER-based study correlated history of breast cancer to better survival of a subsequent epithelial ovarian cancer. However, our results showed that a prior malignancy was associated with a significant negative effect on the overall survival of CRC patients. The disparity between the results of our study and the British study could be attributed to its small sample size [15]. The significantly superior colorectal cancer-specific survival in patients who had a prior history of non-leukemic cancer, along with the results of the above mentioned studies testing other cancers, suggest that –in many cases- the survival of patients with second primary cancer is dependent on histology and tissue of origin of the second primary cancer rather than the prior history of malignancy.

As treatment options of cancer advance and the population age, the number of patients with prior malignancies tends to increase. Many ongoing clinical trials on stage IV CRC have been excluding patients with a prior cancer [16, 17]. The reason behind this exclusion is to ensure the safety of patients, and the assumption that a previous malignancy can potentially interfere with the outcomes of the trial by affecting the overall survival of participants [5, 18, 19]. However, such exclusion may limit the generalizability of the results [20–22]. Moreover, studies on metastatic colorectal cancer, acute myelogenous leukemia, and breast cancer patients have shown that participants of clinical trials show longer survival than non-participants receiving similar treatment. In harmony with that, a previous SEER report has found that 3.5 to 36.9% of cancer patients, diagnosed between 2009 to 2013, has had a prior malignancy [21]. They recommended that more studies should be conducted to understand the impact of prior malignancies on the survival of subsequent ones. Furthermore, they have concluded that 15% of the older population of colorectal cancer patients had a prior malignancy. Although our study found that only 7% of cancer patients had a prior malignancy, the discrepancy could be attributed to the more restrictive inclusion criteria of our study and the inclusion of only subsequent first order colorectal cancer patients. Therefore, the aforementioned studies assert that more patients with a history of prior malignancies should be enrolled in clinical trials [23–25].

To study this point, and assess the validity of such exclusion criterion, we performed a subgroup survival analysis of stage IV CRC patients. According to our results, the exclusion of stage IV CRC patients from clinical trials may be justified if the prior malignancy was leukemia; a prior leukemia diagnosis had a detrimental impact on overall survival of stage IV CRC patients. However, we found that this detrimental effect of history of prior non-leukemic malignancy did not hold true in stage IV CRC patients.

Additionally, the adjusted analysis of overall survival according to the latency period between diagnosis of stage IV CRC and the prior malignancy showed that stage IV CRC was associated with a worse survival only when diagnosed within 7–12 months of the prior malignancy. Whereas, a prior malignancy that was diagnosed before a year or more was not associated with a statistically significant difference in survival.

Our results assert the recommendation that colorectal cancer patients who had a prior history of non-leukemic malignancy should enroll in clinical trials regardless of the stage of the subsequent CRC. In Harmony with that, participants of clinical trials have a better prognosis than non-participants receiving equivalent treatment, and patients shouldn’t be deprived of that benefit as the aforementioned data have shown. Previous studies investigated advanced stages of other cancers and reached similar conclusions; a previous study suggested that a prior malignancy did not negatively affect overall / lung-cancer specific survival in stage IV lung cancer patients [5]. Another study assessing the potential impact of a prior cancer on the mortality of subsequent prostate cancer found that patients with high grade or locally invasive prostate cancer are 50% more likely to die from prostate cancer itself than from the prior malignancy [26]. Moreover, another study investigated the impact of a prior malignancy and found it to have no significant effect on the survival of a subsequent early-stage lung cancer [27].

Recent studies have confirmed the correlation between the side of CRC (left vs. right) and overall survival. Therefore, we analyzed the impact of a prior history of malignancy on the survival of RCRC and LCRC separately [28, 29]. We found that having a history of prior malignancy affected the overall survival and colorectal cancer-specific survival of both LCRC and RCRC in the same pattern as the whole colorectal population. In addition, when we compared stage IV LCRC with/without a prior history of malignancy vs. stage IV RCRC with/ without a prior history of cancer, we found that all LCRC populations, whether with or without prior history of malignancy, had better overall survival compared to RCRC populations. These results support the recent literature evidence showing that LCRC has better survival than RCRC. The results may also highlight the fact that the site of origin of the colorectal cancer is an important contributing factor to survival even for patients with a prior history of cancer. The possible explanation for this is that patients’ outcomes may be driven by colorectal cancer rather than any previous malignancy.

Our study has some limitations associated with using the SEER database. These include missing chemotherapy data and reporting of comorbidities which is problematic, as these are two very important prognostic variables. Other limitations include the unreliable reporting of cause of death in case of multiple primaries [30], the unreliable coding for rare histologies and lacking data on CRC biomarkers, hence we could not assess biomarker information, and lacking data on molecular subtypes of colorectal cancer. Moreover, patients’ data may be lost from the database when they migrate out of SEER registry geographical catchment areas and that may lead to underreporting of second malignancies [31]. Lynch syndrome was not studied in this article due to the lack of data on genetic testing in the SEER database. It occurs only in 1–3% of all CRC cases [32]. However, the 6-month latency period excludes Lynch syndrome synchronous malignancies. A history of endometrial cancer occurred in about 14.7% of our included cases, while the rest of reported CRC prior malignancies are not commonly associated with Lynch syndrome.

## Conclusion

We recommend that colorectal cancer patients who had a prior history of non-leukemic malignancy should enroll in clinical trials without concerns of the prior malignancy affecting their outcomes, and regardless of the stage or the side of the subsequent CRC. This did not apply only for stage IV CRC patients who were diagnosed within 7–12 months of the prior malignancy where the presence of a prior malignancy significantly worsened outcomes.

## Additional files

Additional file 1: All-cause (a) and colon cancer-specific (b) survival of right colon cancer, and all-cause (c) and colon cancer-specific (d) survival of left colon cancer. (JPG 1636 kb)

Additional file 2: All-cause survival of stage IV right colon cancer (a), stage IV left colon cancer (b), and stage IV right vs. left colon cancer (c). (JPG 836 kb)

## Data Availability

The datasets generated and/or analysed during the current study are available in the SEER database, https://seer.cancer.gov/data/.

## Abbreviations

CRC: Colorectal cancer
HR: Hazard ratio
LCRC: Left colon cancer
RCRC: Adenomatous polyposis coli
RCRC: Carbohydrate antigen 19–9
RCRC: Carbohydrate antigen 72–4
RCRC: Microsatellite instability
RCRC: Polymerase chain reaction
RCRC: Right colon cancer
RCRC: Septin 9
RCRC: Serpentine
SEER: The Surveillance, Epidemiology, and End Results
