# Supplementary figures and images for: Impact of prior malignancies on outcome of colorectal cancer; revisiting clinical trial eligibility criteria
Source: BMC Cancer. 2019 Aug 30;19:863. doi: 10.1186/s12885-019-6074-6 (PMC6716811; doi:10.1186/s12885-019-6074-6)

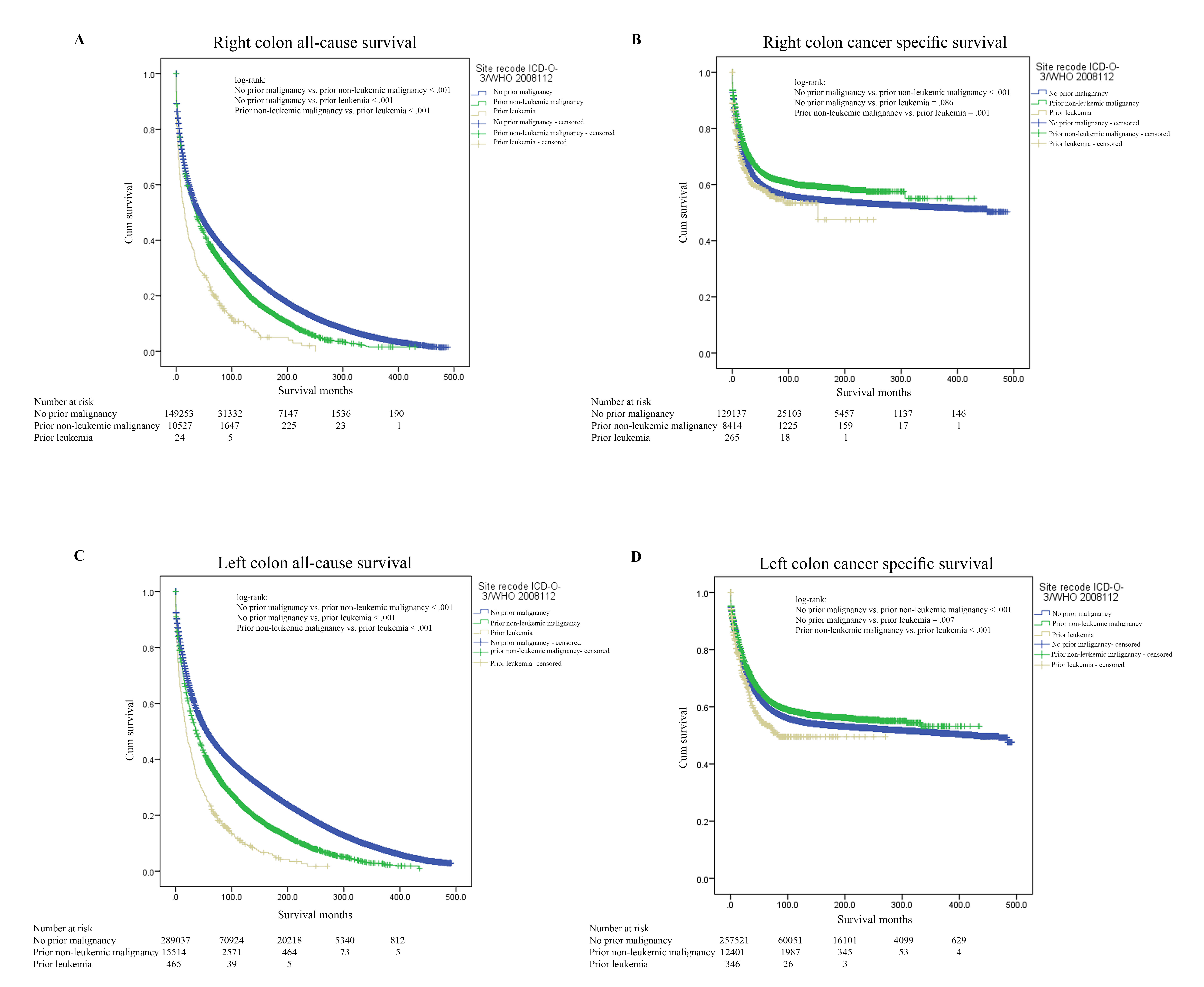

Supplement: Supplementary file 1 — All-cause (a) and colon cancer-specific (b) survival of right colon cancer, and all-cause (c) and colon cancer-specific (d) survival of left colon cancer. (JPG 1636 kb) [file 12885_2019_6074_MOESM1_ESM.jpg]

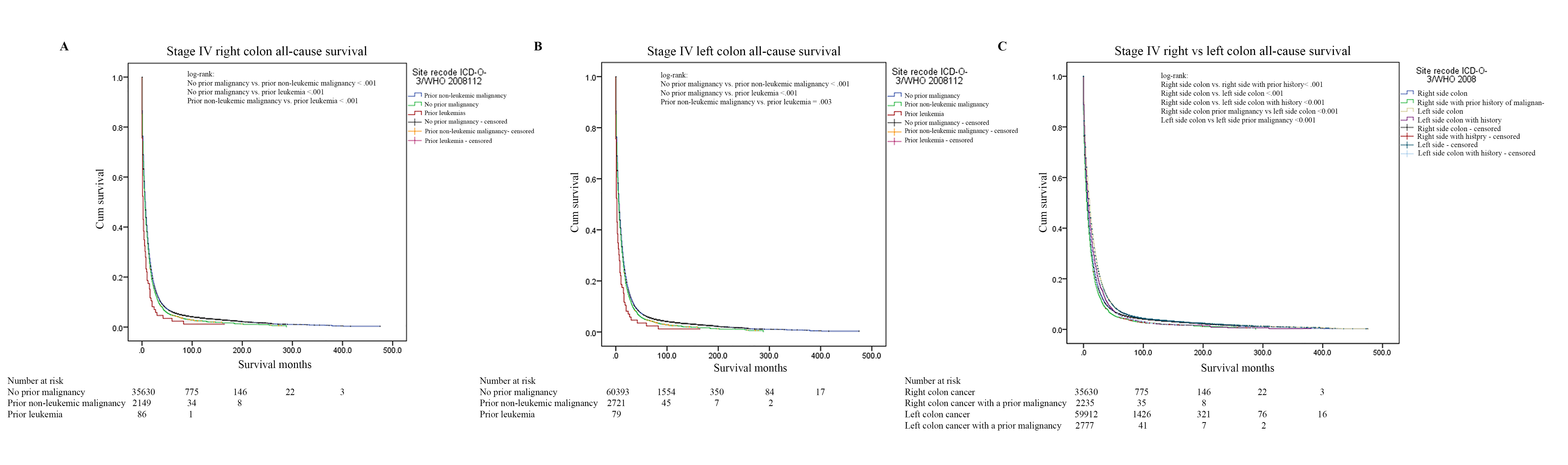

Supplement: Supplementary file 2 — All-cause survival of stage IV right colon cancer (a), stage IV left colon cancer (b), and stage IV right vs. left colon cancer (c). (JPG 836 kb) [file 12885_2019_6074_MOESM2_ESM.jpg]
